# P-1068. Cluster of Stenotrophomonas maltophilia Bacteremia in Hemodialysis Patients: Hygiene Under Scrutiny

**DOI:** 10.1093/ofid/ofaf695.1263

**Published:** 2026-01-11

**Authors:** Julio Cruz, Katherine Rodriguez, Karina Espaillat, Ana Fernandez, Xiomara Rodriguez, Claudia Blanco, Talia Flores

**Affiliations:** Hospital Salvador B. Gautier, Santo Domingo, Distrito Nacional, Dominican Republic; Hospital Salvador B. Gautier, Santo Domingo, Distrito Nacional, Dominican Republic; Hospital Salvador B. Gautier, Santo Domingo, Distrito Nacional, Dominican Republic; Hospital Salvador B. Gautier, Santo Domingo, Distrito Nacional, Dominican Republic; Hospital Salvador B. Gautier, Santo Domingo, Distrito Nacional, Dominican Republic; SDI, Santo Domingo, Distrito Nacional, Dominican Republic; Hospital Salvador B. Gautier, Santo Domingo, Distrito Nacional, Dominican Republic

## Abstract

**Background:**

Catheter-related bloodstream infections (CRBSIs) contribute significantly to morbidity and mortality in patients undergoing hemodialysis. *Stenotrophomonas maltophilia* is an opportunistic, multidrug-resistant, Gram-negative non-fermenting bacillus increasingly implicated in nosocomial infections. We report a cluster of *S. maltophilia* bacteremia in hemodialysis patients, highlighting the need for stringent infection control measures.

**Figure:**
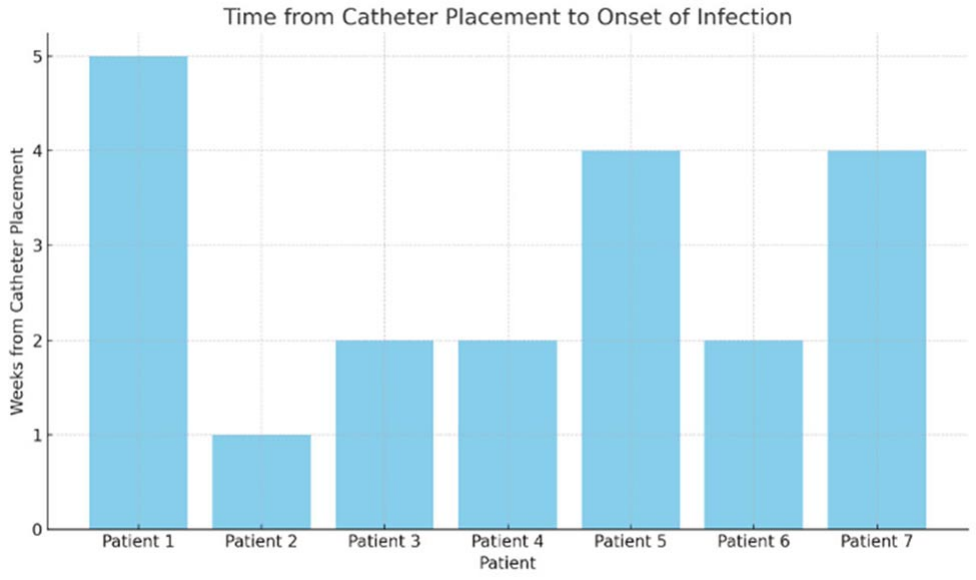
This bar chart displays the time interval, in weeks, between central venous catheter placement and the onset of bacteremia in seven hemodialysis patients. The time to infection varied from 1 to 5 weeks post-catheter insertion. This variability suggests that the infections were more likely due to ongoing contamination or inadequate hygiene during catheter care, rather than isolated insertion-related events. These findings support the hypothesis of a persistent healthcare-associated source related to suboptimal vascular access practices

**Figure:**
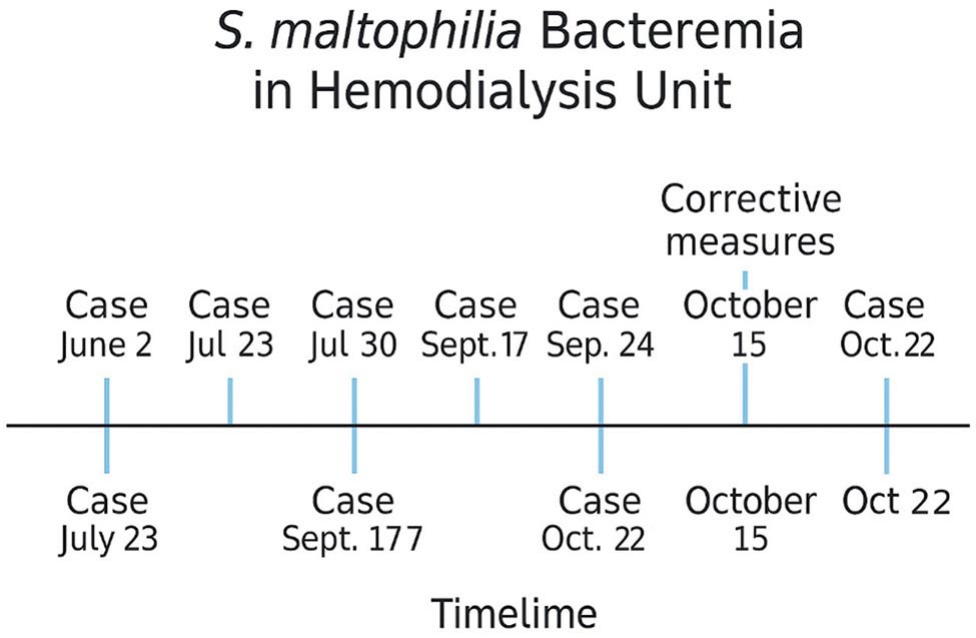
The timeline illustrates the sequential occurrence of seven cases of Stenotrophomonas maltophilia bacteremia in a hemodialysis unit between June 2 and October 22. Two peaks of incidence were observed: one in July (two cases) and another between September and October (three cases), suggesting progressive transmission. Corrective measures—including reinforcement of vascular access hygiene and staff retraining—were implemented on October 15. No new cases were reported after these interventions, indicating that the outbreak was likely controlled through improved infection prevention practices.

**Methods:**

We identified seven patients with end-stage renal disease receiving hemodialysis at our hospital center who developed *S. maltophilia* bacteremia over a defined period. Clinical records were reviewed for demographic data, clinical presentation, microbiological findings, management, and outcomes. Blood cultures were processed using standard microbiologic techniques, and susceptibility testing was performed according to CLSI guidelines.

**Figure:**
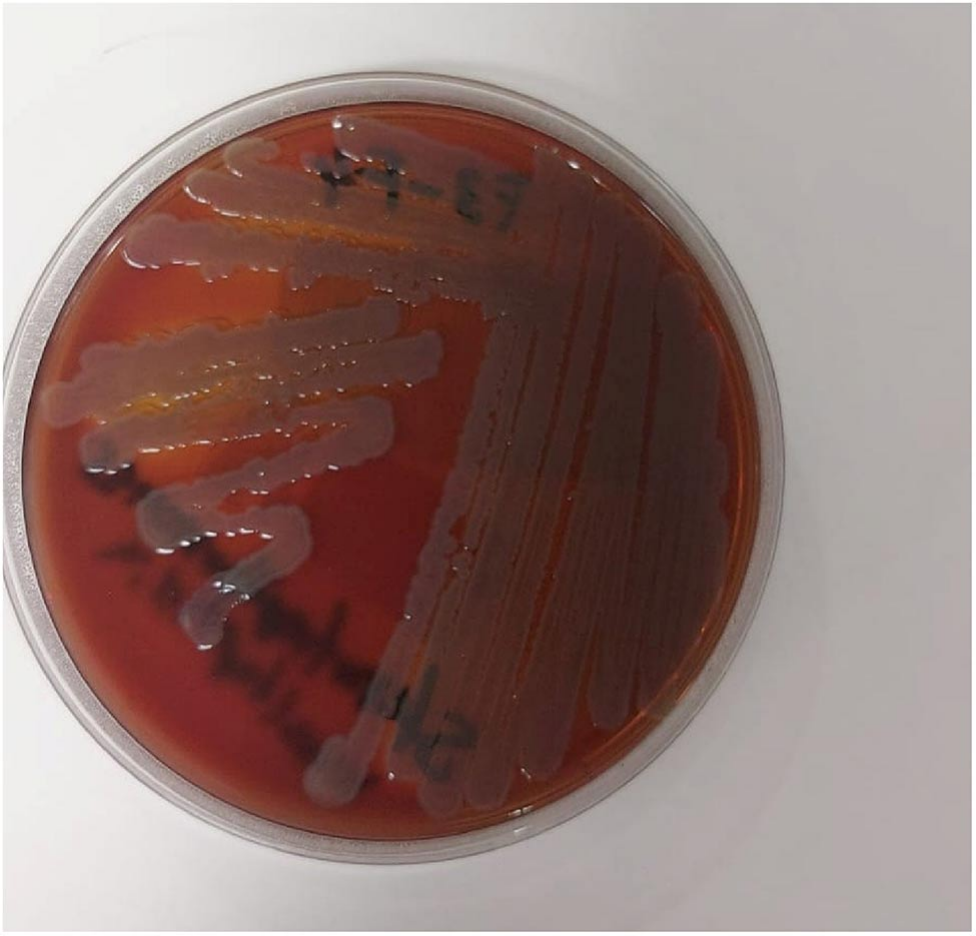
Stenotrophomonas maltophilia on blood agar plate Visible colonies of Stenotrophomonas maltophilia on blood agar from a peripheral blood sample of one patient

**Results:**

Seven cases (five male, two female), aged 41 to 67 years, presented with fever, chills, malaise, and fatigue during or shortly after dialysis sessions. Blood cultures confirmed *S. maltophilia* bacteremia in all cases. The infection prompted removal of dialysis catheters and administration of targeted antibiotic therapy based on susceptibility results, primarily with levofloxacin and trimethoprim-sulfamethoxazole. All patients responded favorably to treatment. A review of dialysis unit practices revealed lapses in vascular access hygiene, prompting immediate corrective measures. No additional cases were identified after implementing enhanced infection prevention protocols.

**Conclusion:**

This cluster of *S. maltophilia* bacteremia underscores the importance of rigorous vascular access hygiene and infection control in dialysis units. Prompt recognition, targeted antimicrobial therapy, and systemic changes in practice were crucial in controlling the outbreak and preventing recurrence. Active surveillance and continuous quality improvement are essential in high-risk settings such as hemodialysis centers.

**Disclosures:**

All Authors: No reported disclosures

